# Magnetic properties of biofunctionalized iron oxide nanoparticles as magnetic resonance imaging contrast agents

**DOI:** 10.3762/bjnano.10.193

**Published:** 2019-10-02

**Authors:** Natalia E Gervits, Andrey A Gippius, Alexey V Tkachev, Evgeniy I Demikhov, Sergey S Starchikov, Igor S Lyubutin, Alexander L Vasiliev, Vladimir P Chekhonin, Maxim A Abakumov, Alevtina S Semkina, Alexander G Mazhuga

**Affiliations:** 1 Shubnikov Institute of Crystallography of FSRC “Crystallography and Photonics” RAS, 119333, Moscow, Russia; 2 Lebedev Physical Institute, Russian Academy of Sciences, 119991, Moscow, Russia; 3 Moscow State University, Physical Department, 119992, Moscow, Russia; 4 National Research Center "Kurchatov Institute", 123182, Moscow, Russia; 5 Moscow Institute of Physics and Technology (State University), MIPT, 141701 Moscow Region, Russia; 6 Department of Medical Nanobiotechnology, Pirogov Russian National Research Medical University, Moscow, Russia; 7 Laboratory of Biomedical Nanomaterials, NUST MISiS, Moscow Russia; 8 Mendeleev Chemical Technological University, Moscow, Russia

**Keywords:** iron oxides, Mössbauer spectroscopy, MRI contrast agents, nanocrystalline materials, NMR spectroscopy, Raman spectroscopy

## Abstract

**Background:** One of the future applications of magnetic nanoparticles is the development of new iron-oxide-based magnetic resonance imaging (MRI) negative contrast agents, which are intended to improve the results of diagnostics and complement existing Gd-based contrast media.

**Results:** Iron oxide nanoparticles designed for use as MRI contrast media are precisely examined by a variety of methods: powder X-ray diffraction (XRD), transmission electron microscopy (TEM), Raman spectroscopy, Mössbauer spectroscopy and zero-field nuclear magnetic resonance (ZF-NMR) spectroscopy. TEM and XRD measurements reveal a spherical shape of the nanoparticles with an average diameter of 5–8 nm and a cubic spinel-type crystal structure of space group *Fd*−3*m*. Raman, Mössbauer and NMR spectroscopy clearly indicate the presence of the maghemite γ-Fe_2_O_3_ phase. Moreover, a difference in the magnetic behavior of uncoated and human serum albumin coated iron oxide nanoparticles was observed by Mössbauer spectroscopy.

**Conclusion:** This difference in magnetic behavior is explained by the influence of biofunctionalization on the magnetic and electronic properties of the iron oxide nanoparticles. The ZF-NMR spectra analysis allowed us to determine the relative amount of iron located in the core and the surface layer of the nanoparticles. The obtained results are important for understanding the structural and magnetic properties of iron oxide nanoparticles used as *T*_2_ contrast agents for MRI.

## Introduction

Nowadays, magnetic nanoparticles (MNPs) are widely used in biology and medicine. A large number of studies [1–4] have shown different prospects of their use for sample preparation, in genomic and proteomic analysis [5], for drug delivery [6], as magnetic resonance imaging (MRI) contrast agents [7], and for magnetic hyperthermia [8]. This wide variety of applications is due to the unique combination of magnetic, optical and chemical properties that are characteristic of MNPs. However, the structure and composition of the particular magnetic material strongly influences the behavior of the nanoparticles. Thus, they should be precisely studied before being used in biology and medicine.

MRI is a widely approved procedure used to visualize tissue in vivo and to reveal pathological foci. The contrast between the tissues in MRI images depends on their properties such as fat and water content as well as on the sequence of the procedure parameters. There are three main characteristics that determine the contrast of the image: 1) proton density; 2) the spin–lattice relaxation time *T*_1_; and 3) the spin–spin relaxation time *T*_2_. Generally, these natural differences in tissue properties provide the necessary contrast, but in some cases, the pathological focus cannot be visualized in the images, for instance, due to size effects or the difficulty in delineating boundaries to determine their composition. In such cases, contrast agents should be used.

The currently available contrast agents are divided into two main groups: 1) gadolinium-based positive contrast agents and 2) iron-oxide-based negative contrast agents. Positive contrast agents, which include the most popular agents containing paramagnetic gadolinium, reduce the spin–lattice relaxation time (*T*_1_), which makes the pathological focus brighter. Negative contrast agents typically contain MNPs with Fe^2+^ and Fe^3+^ ions. They reduce the *T*_2_ relaxation time and therefore weaken the signal from the tissues that absorbed the agent. Unlike gadolinium agents, these agents have fewer contraindications for patients with renal failure and are less toxic [9].

However, despite the fact that the iron-containing agents have been approved for medical use, they are considered to have inferior characteristics as compared to positive contrast agents. The properties of iron-containing oxide nanoparticles significantly depend on the synthesis conditions, their size, shape, morphology, crystal and magnetic structure, phase composition and type of coating. Their detailed (and often complex) characterization is required in many cases.

The functionalization of nanoparticles with bovine serum albumin (BSA) and human serum albumin (HSA) is one potential method to enhance their biocompatibility. There are several directions in the development of coating types: coating of the nanoparticles themselves [10], design of BSA microcapsules with iron oxide nanoparticles located inside and outside of each capsule [11] and integration of the nanoparticles in the matrix of HSA threads, as will be discussed in this paper.

The problem of distinguishing between magnetite Fe_3_O_4_ and maghemite γ-Fe_2_O_3_, both of which usually appear as synthesis products of iron oxide nanoparticles, has been repeatedly emphasized, and the exact composition of the MNPs is usually determined using X-ray diffraction (XRD) or Mössbauer spectroscopy with and without magnetic field [12–14].

In this work, we show other options for solving this problem using Raman and nuclear magnetic resonance (NMR) spectroscopy, where the latter provides the most descriptive results.

Traditionally, XRD is one of the most popular methods used to study crystal structure. However, in the case of iron oxides, especially with nonstoichiometric composition, this method does not allow for the precise determination of the structure due to the presence of both magnetite Fe_3_O_4_ and maghemite γ-Fe_2_O_3_.

Another method to distinguish between Fe^2+^ and Fe^3+^ and their positions in the crystal structure is Mössbauer spectroscopy. However, the use of ionizing radiation and radioactive sources in this method limits the possibility of its transfer to production.

Raman spectroscopy can also be used to discriminate nanoscale magnetite and maghemite. However, this method gives only qualitative information about the iron oxide structure and does not allow the number of Fe^2+^ and Fe^3+^ atoms or their positions to be determined.

Another method that makes it possible to evaluate the magnetic structure of the sample is solid-state ^57^Fe NMR. This method avoids the use of ionizing radiation and allows data on the structure of the magnetic sample to be obtained under a magnetic field similar to that used in MRI.

The purpose of this article is to investigate the crystal structure and magnetic properties of iron oxide nanoparticles, which have already been proven to be effective as MRI contrast agents as studied by different techniques, including XRD, Mössbauer, Raman and ^57^Fe NMR spectroscopy. The question of the effect of the type of coating on the particle size and their magnetic properties is also raised. It has been shown that different types of coatings shift the magnetic blocking temperature [10,13–14]. In our present study, we observe a superparamagnetic transition of coated and uncoated samples in the temperature range from 10 to 300 K by means of Mössbauer spectroscopy, which allows one to estimate the ratio of the magnetic and superparamagnetic phases at different temperatures.

## Results and Discussion

**Figure 1:**
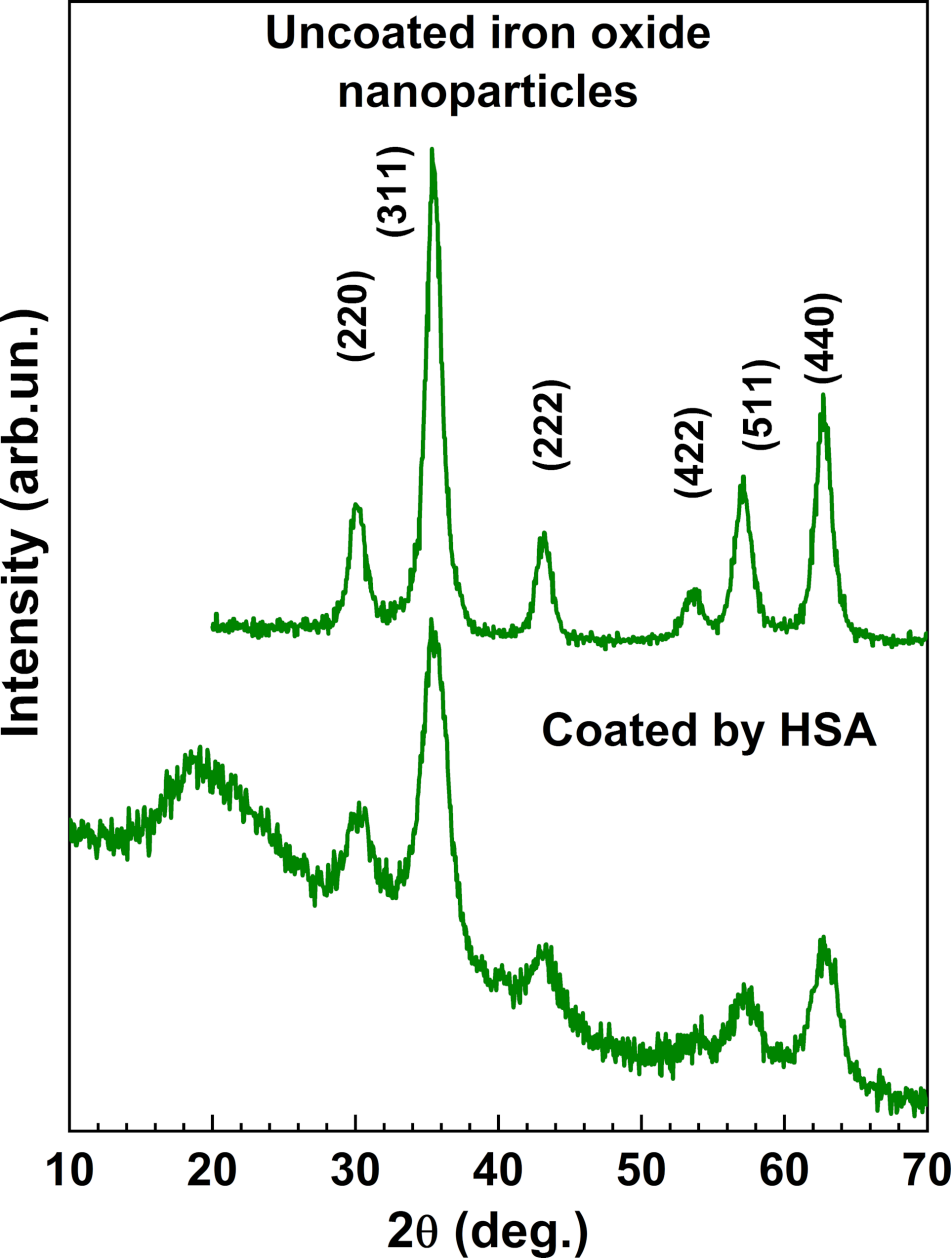
XRD patterns of coated and uncoated magnetic nanoparticles.

The XRD patterns of the nanoparticles are shown in Figure 1. All peaks can be indexed according to a cubic spinel-type crystal structure with a 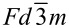 space group, which is typical for magnetite Fe_3_O_4_ or maghemite γ-Fe_2_O_3_ [15–16]. The broad peak at 2θ = 19° and high background level in the pattern of the coated nanoparticles are due to HSA [17].

The mean size of the nanoparticles was estimated by applying the Scherrer equation to the most intensive (311) peak while assuming a spherical shape of the nanoparticles (dimensionless shape factor *K* = 0.94). The coated and uncoated nanoparticles were found to have a diameter of 4.0(2) and 3.5(5) nm, respectively. Note that the Scherrer equation gives the mean size within the coherent X-ray scattering region, and the size can be slightly different from the values obtained by transmission electron microscopy (TEM).

**Figure 2:**
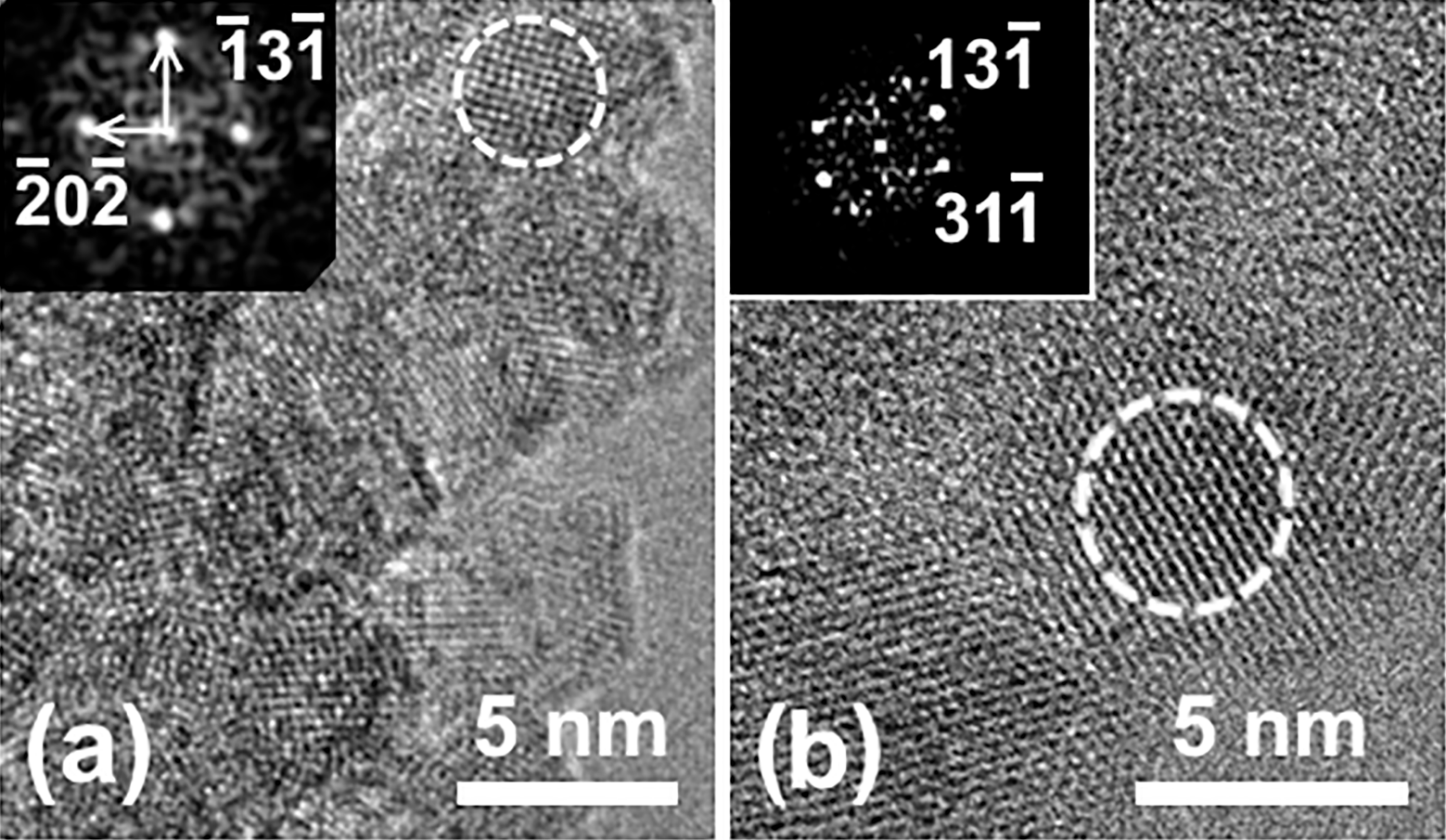
HRTEM images of uncoated (a) and HSA-functionalized samples (b).

**Figure 3:**
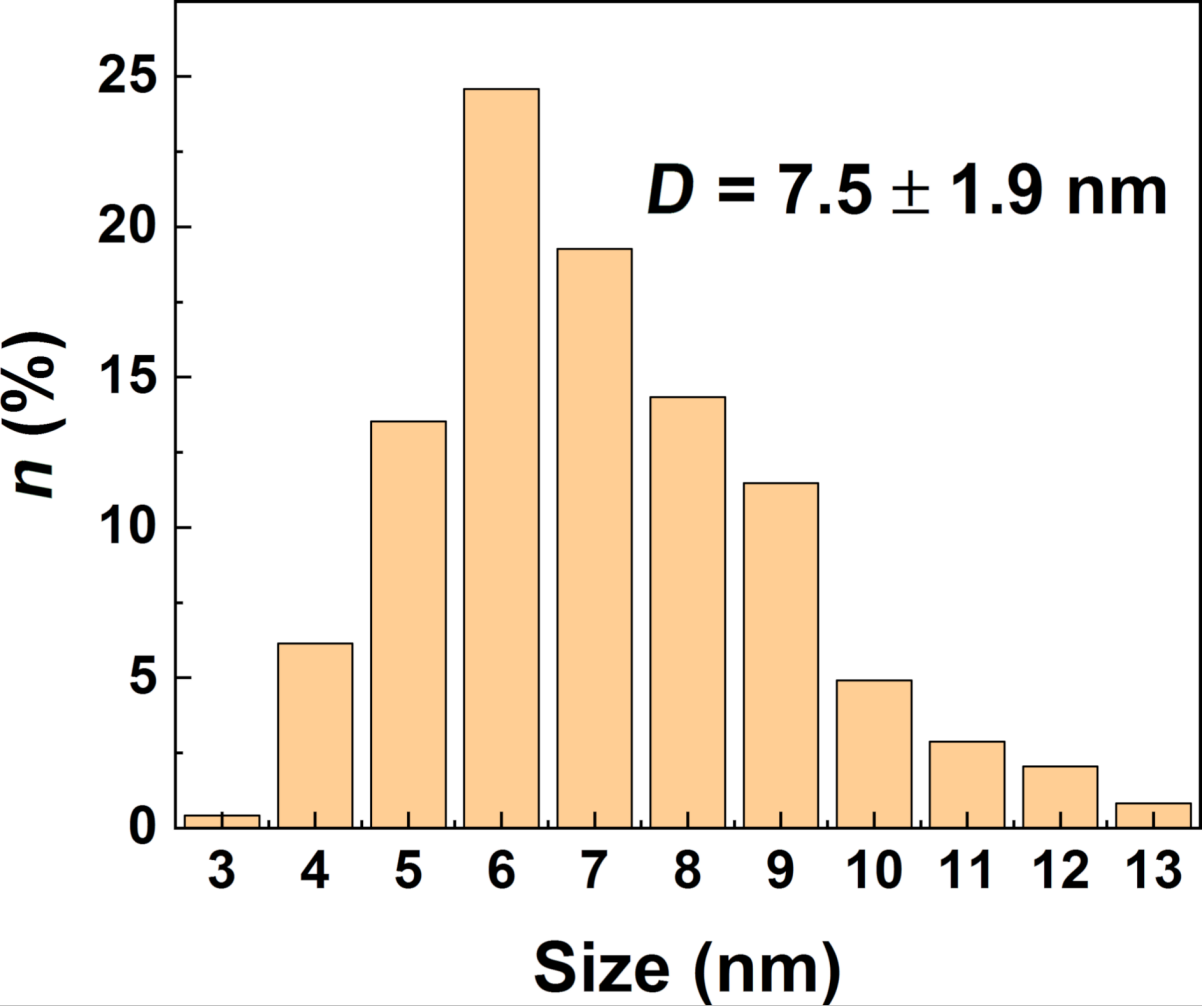
The particle size distribution estimated from the HRTEM images in Figure 2.

The TEM images of the nanoparticles are presented in Figure 2. The particle size distribution estimated from the high-resolution TEM (HRTEM) images is shown in Figure 3. Roughly 70% of the particles are of 5–8 nm in diameter (half maximum of the size distribution) and all of them exhibit an equiaxed morphology. The analysis of the electron diffraction pattern along with the fast Fourier transform (FFT) patterns (see insets in Figure 2a and Figure 2b) indicates that the iron oxide nanoparticles adopted a diamond-type cubic crystal lattice structure (space group 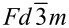) that is typical for magnetite (Fe_3_O_4_) [15] and/or disordered maghemite (γ-Fe_2_O_3_) [16]. The HRTEM images of several selected particles in both samples were analyzed by direct measurements of the lattice spacing and angles between the crystal planes and the inspection of FFT patterns. Two examples of the analysis are presented in Figure 2a and Figure 2b, where the outlined particles were observed in the [323] and [114] zone axis, respectively. The lattice parameters obtained for the particles selected in Figure 2 are very similar (*a* = 0.84 nm), and hence magnetite (Fe_3_O_4_) and disordered maghemite γ-Fe_2_O_3_ compounds cannot be resolved by HRTEM in such images.

**Figure 4:**
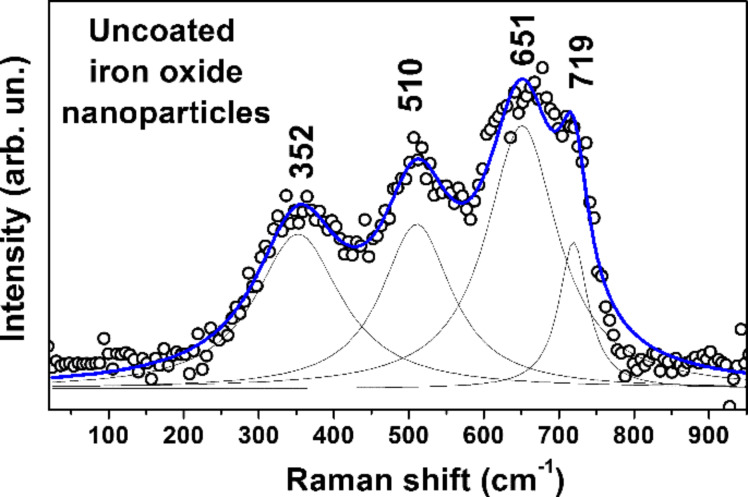
Raman spectrum of uncoated nanoparticles. Fitting of the peaks in the region up to 950 cm^−1^ is shown. The peaks at 352, 510, 651, and 719 cm^−1^ correspond to the oxygen vibrations in maghemite γ-Fe_2_O_3_. The solid lines are the approximation of the experimental spectrum by Gaussian distribution.

The Raman spectrum of the uncoated nanoparticles is shown in Figure 4, and the fitting of the peaks in the frequency region up to 950 cm^−1^ is presented. The spectrum can be decomposed into four main peaks centered at 352, 510, 651, and 719 cm^−1^.

According to [18], Raman peaks at about 377, 510, 670, and 715 cm^−1^ are characteristic of the maghemite phase, whereas typical peaks of magnetite would be expected at about 310, 540 and 670 cm^−1^ [18–19]. The peaks at 310 and 540 cm^−1^ are not resolved in the experimental spectrum of Figure 4, and the most intensive peak of magnetite at about 670 cm^−1^ (if present) might overlap with the maghemite peak and increase the overall intensity in this frequency range.

Finally, a comparative analysis of the intensity and position of the Raman lines indicates that the amount of magnetite is negligible compared to maghemite γ-Fe_2_O_3_.

Since the Raman studies of the HSA-coated nanoparticles revealed luminescence from the HSA organic compounds, we were not able to record the Raman spectrum of these particles using a laser at a wavelength of 671 nm.

**Figure 5:**
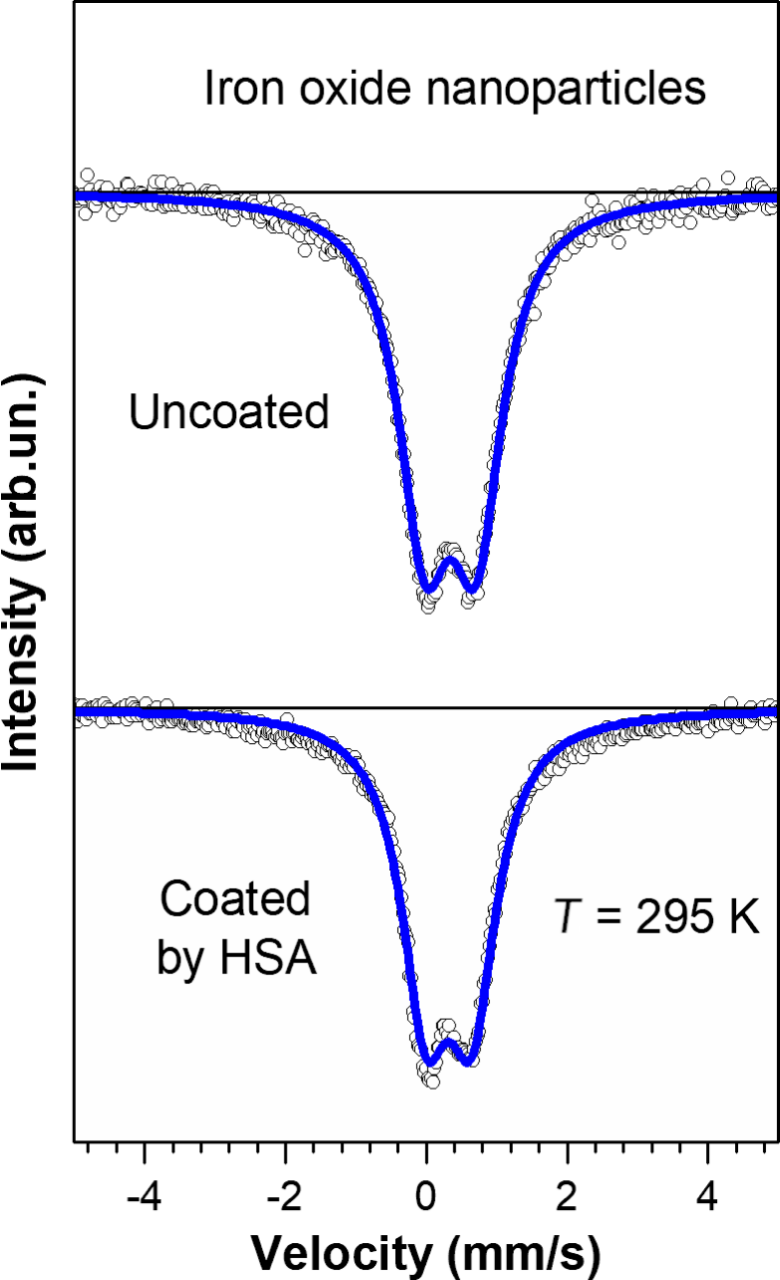
Room temperature Mössbauer spectra of HSA-coated magnetic nanoparticles and uncoated particles. The solid lines are a fit to the experimental data.

The Mössbauer spectra of the HSA-coated MNPs and uncoated MNPs at room temperature are shown in Figure 5. Both spectra look similar and can be described by a single paramagnetic component (doublet) with hyperfine parameters: isomer shift δ = 0.32(1) mm/s, quadrupole splitting Δ = 0.66(2) mm/s for HSA-coated MNPs and δ = 0.34(1) mm/s, Δ = 0.74(1) mm/s for uncoated MNPs. These values correspond to Fe^3+^ ions in the high spin state.

It is worth mentioning that bulk magnetite and maghemite both have a very high Curie temperature of about 860 and 950 K, respectively. However, small single-domain MNPs with a diameter of less than 10 nm are highly sensitive to thermal energy. Even room temperature is sufficient to destabilize the magnetic moment of the entire nanoparticle and transfer it to a paramagnetic state.

**Figure 6:**
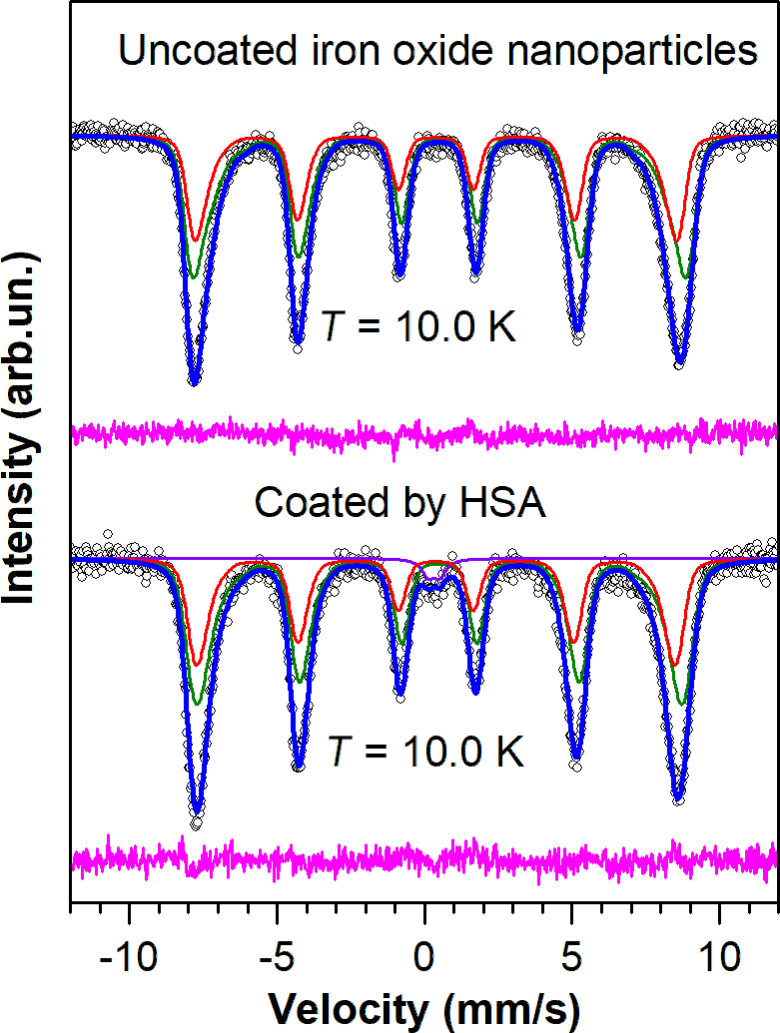
Mössbauer spectra of uncoated and HSA-coated magnetic nanoparticles at 10 K. Red and green sextets correspond to iron ions in A- and B-sites of the spinel crystal structure.

The ^57^Fe Mössbauer spectra of iron oxide nanoparticles obtained at 10 K are shown in Figure 6. The magnetic hyperfine splitting observed in the spectra indicates a magnetically ordered state of the iron ions. A slight broadening of the lines found in the inner part of the spectrum is due to thermal fluctuations of the iron magnetic moments, which is quite usual for spectra of nanoscale iron oxides.

According to the XRD and TEM data, the iron oxide in the sample adopted a cubic spinel crystal structure, which is characteristic of magnetite (Fe_3_O_4_) and maghemite (γ-Fe_2_O_3_). In these crystals, iron ions occupy two nonequivalent sites, tetrahedral (A-site) and octahedral [B-site]. The cationic distribution can be represented as: (Fe^3+^)_A_[Fe^2.5+^_2_]_B_O_4_ in the case of magnetite and (Fe^3+^)_A_[Fe^3+^_5/3_^□^_1/3_]_B_O_4_ for maghemite, where ^□^ denotes vacancies. The fast electron exchange between the Fe^3+^ and Fe^2+^ ions in the B-sites of magnetite leads to an average valence Fe^2.5+^.

At about *T*_V_ = 120 K the Verwey transition occurs, which leads to changes in the magnetite crystal structure, as well as the electronic and magnetic properties [20]. Below *T*_V_ the electron exchange between the Fe^3+^ and Fe^2+^ ions in the B-sites is frozen. These changes can be easily identified in the Mössbauer spectrum of magnetite. Mössbauer spectroscopy is a highly sensitive method with respect to the valence of iron ions in these bulk oxides, since the hyperfine parameters corresponding to Fe^3+^ and Fe^2+^ ions are well distinguishable in the spectra.

**Figure 7:**
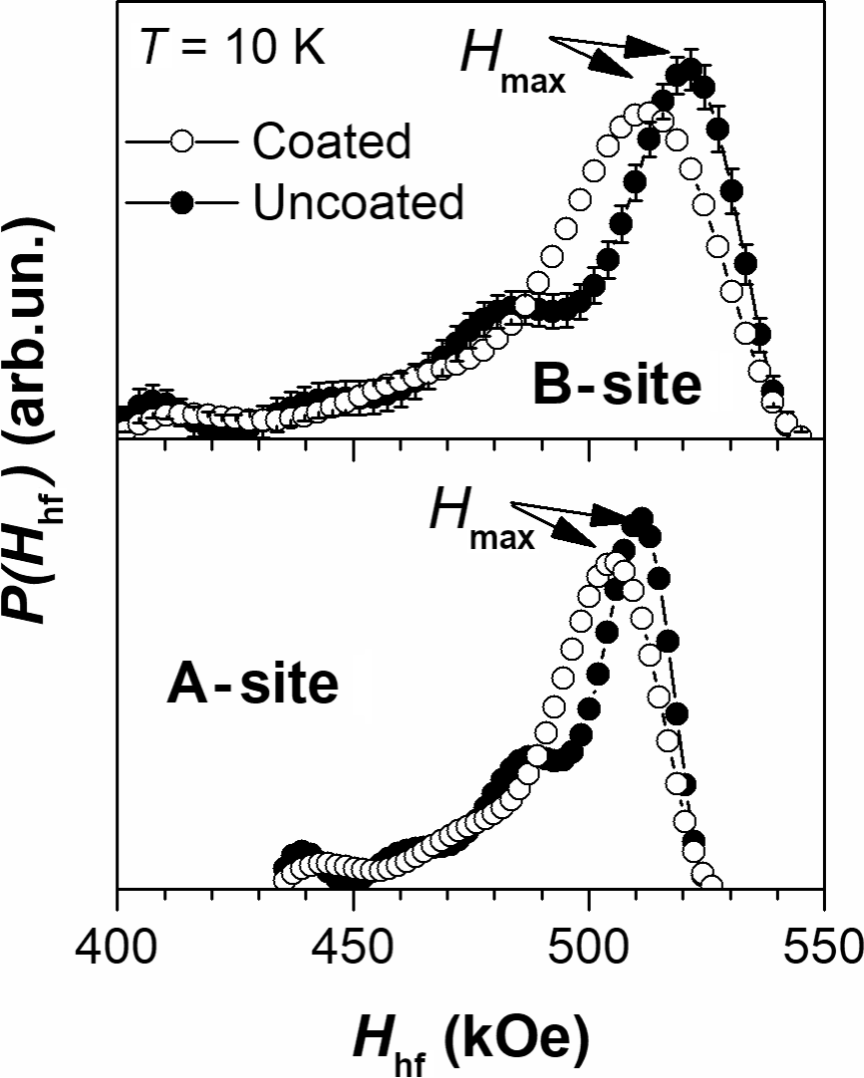
Distribution of the magnetic field *H*_hf_ values obtained from the Mössbauer spectra for uncoated and HSA-coated magnetic nanoparticles.

For the processing of the low-temperature Mössbauer spectra of uncoated MNPs, a model consisting of two magnetic components (sextets) was used (Figure 6). Each sextet corresponds to nonequivalent states of iron ions in the A- and B-sites. The values of the magnetic hyperfine field, *H*_hf_, in the sextets were described by certain distribution functions, *P*(*H*_hf_), as shown in Figure 7. The shape of *P*(*H*_hf_) for both components was assumed to be similar. The ratio of the components was fixed in the first approximation at a value of 1.67, which is characteristic of the ratio of iron ions in B- and A-sites in γ-Fe_2_O_3_ and then fixed at a value of 2.0, which is characteristic of magnetite Fe_3_O_4_. The distribution function in *H*_hf_ values is used for a number of reasons, such as the size distribution of the nanoparticles, the different nature of the interparticle interaction, lattice defects and surface effects [21].

According to Table 1 and the data of [22], the obtained values of the isomer shift, δ, quadrupole shift, ε, and *H*_hf_ correspond to the high spin state of Fe^3+^ iron ions in maghemite γ-Fe_2_O_3_ [14]. When attempting to process the Mössbauer spectra taking into account the 1:2 ratio of the components (as it is expected in magnetite), we obtained the same values of isomer shifts and a slightly changed distribution of *H*_hf_. The component corresponding to Fe^2+^ ions in Fe_3_O_4_ was not detected, and therefore, we presume that the γ-Fe_2_O_3_ phase dominates in these particles. This fact correlates with the observation of [14] where it was found that smaller (<11 nm in diameter) nanoparticles preferably adopt a maghemite phase.

**Table 1:** Hyperfine parameters for two magnetic sextets calculated from the Mössbauer spectra at 10 K. *H*_hf_ is the magnetic hyperfine field at iron nuclei, *H*_max_ is the value at the maximum of the field in the distribution function *P*(*H*_hf_) shown in Figure 7, ε is the quadrupole shift, and δ is the isomer shift. The calculation was based on the A- and B-site occupation by Fe^3+^ at a ratio of 1:1.67, which is characteristic of maghemite.

No.	δ, mm/s	ε, mm/s	µ_0_*H*_max_, T	Site
				
Uncoated MNPs at 10 K				

1	0.39(1)	0.00	51.2(2)	Fe^3+^ (A) site
2	0.50(1)	0.00	52.3(2)	Fe^3+^ [B] site
				
HSA-coated MNPs at 10 K				

1	0.38(1)	0.00	50.6(1)	Fe^3+^ (A) site
2	0.50(1)	0.00	51.4(1)	Fe^3+^ [B] site

**Figure 8:**
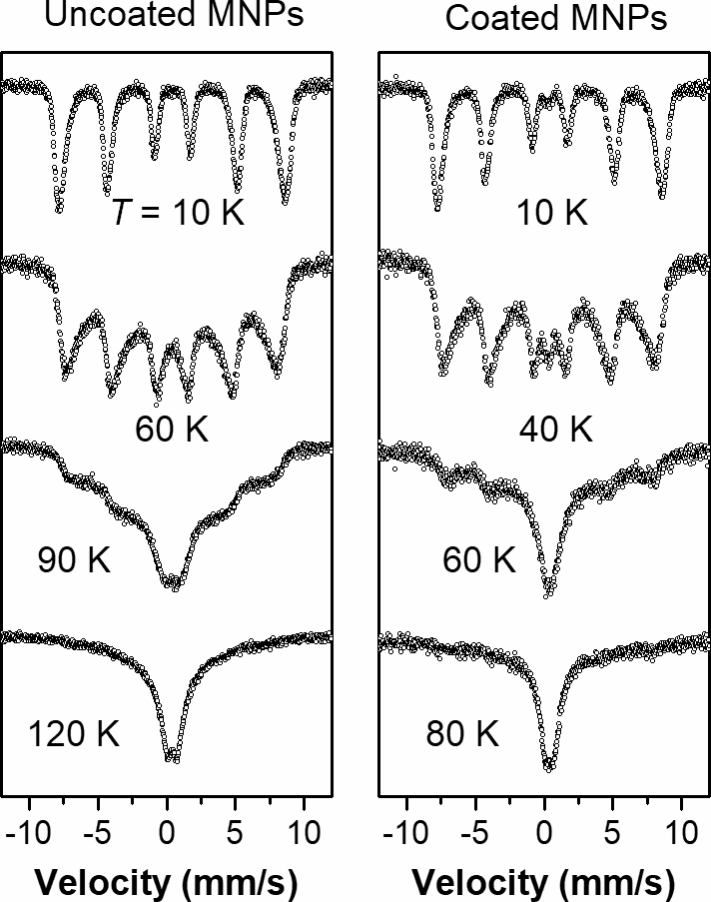
Mössbauer spectra for uncoated and HSA-coated magnetic nanoparticles (MNPs) at temperature ranging from 10 to120 K. The superparamagnetic transition in uncoated nanoparticles is observed at a higher temperature than in coated nanoparticles.

The Mössbauer spectra of uncoated and HSA-coated MNPs at higher temperatures are shown in Figure 8. A typical superparamagnetic behavior was observed in the spectra of both samples. However, the transition to the paramagnetic state occurs at a lower temperature (80 K) in coated MNPs than in uncoated MNPs (120 K).

As follows from Figure 7, Figure 8 and Table 1, there are several significant differences in the Mössbauer spectra of uncoated and HSA-coated MNP samples. The shape of the field distribution function *P*(*H*_hf_) obtained at 10 K is different for uncoated and coated particles. In particular, there is a decrease in the maximum field, *H*_max_, in the spectrum of the coated sample compared to the uncoated one. The observed transition temperature from the magnetically ordered to the paramagnetic state is lower for the coated nanoparticles compared to the uncoated nanoparticles [23–24].

According to XRD and TEM, the HSA-coated and uncoated nanoparticles have a similar size, shape, crystal structure and phase composition. Since the nonmagnetic HSA proteins separate the coated particles from each other, the magnetic interaction between the particles is weaker. Therefore, the magnetic moment fluctuates somewhat more for the coated compared to the uncoated particles. This leads to a decrease in the *H*_max_ value, the appearance of a doublet at a lower temperature and a transition to the paramagnetic state at a lower temperature in the HSA-coated nanoparticles as observed in [10] with Au coating. A similar temperature evolution of the Mössbauer spectra was also observed in [25–26].

**Figure 9:**
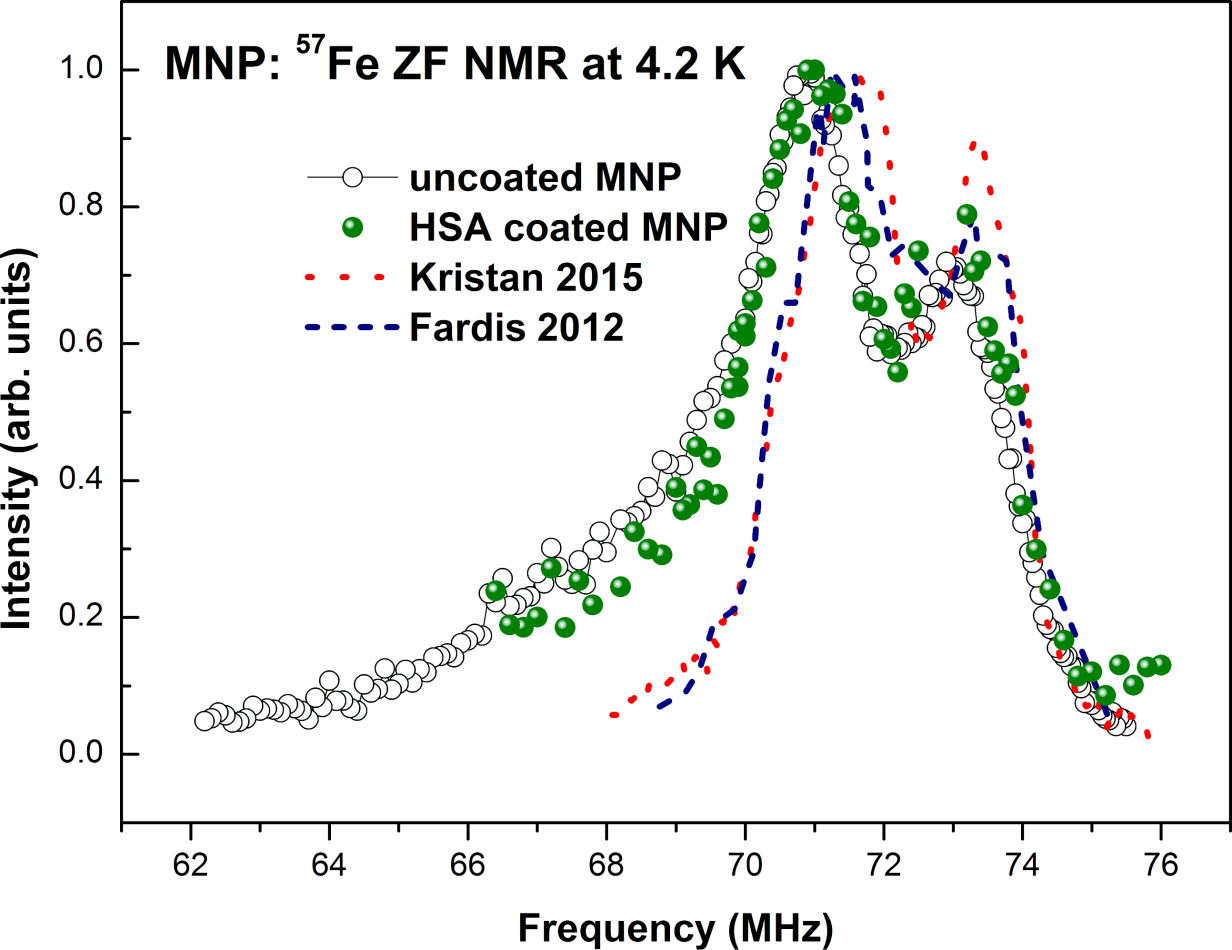
Experimental ZF-NMR spectrum of ^57^Fe nuclei measured at 4.2 K in our metal nanoparticles (MNPs). Open circles and green spheres correspond to our samples of the uncoated and HSA-coated nanoparticles, respectively. The blue dashed line is the spectrum of maghemite nanoparticles adopted from [27]; the red dashed line is the spectrum of maghemite nanoparticles adopted from [28].

Based on the Mössbauer data, we calculated the expected frequency of the NMR signal and conducted zero-field NMR (ZF-NMR) measurements. The NMR spectra of ^57^Fe nuclei in uncoated and HSA-coated samples measured at zero external magnetic field at 4.2 K are shown in Figure 9. The spectra demonstrate a very broad intensity distribution in the range from approximately 62–76 MHz and contain two distinct peaks at 70.9 and 73.0 MHz and a broad low frequency shoulder in the frequency range of 64–69 MHz. The spectra can be successfully decomposed into three Gaussian peaks with center positions at 70.92 and 73.11 MHz for the two sharp peaks and at about 69.50 MHz for the low frequency shoulder line. The fitted parameters for the NMR and Mössbauer spectra are given in Table 2.

**Table 2:** Parameters of the NMR and Mössbauer spectra calculated from experimental data for the uncoated sample. The center frequency, the integral intensity and the line width are the parameters of the Gaussian approximation of the NMR spectra; the local magnetic field values *H*_loc_ at the Fe site were extracted from the experimental ZF-NMR ^57^Fe spectrum measured at 4.2 K, (Figure 9) and *H*_max_ is the field obtained from the Mössbauer measurements at 10 K.

	ZF-NMR				Mössbauer	
			
No.	Center frequency (MHz)	Integral intensity (%)	Linewidth (MHz)	µ_0_*H*_loc_ (T)	µ_0_*H*_max_ (T)	*H*_loc_^NMR^/*H*_max_^MB^
						
1	70.92(1)	26.8(1)	1.42(4)	51.54(1)	51.2(2)	1.01
2	73.10(1)	22.5(2)	1.39(5)	53.13(1)	52.3(2)	1.02
3	69.50(2)	50.7(5)	4.70(30)	50.51(2)	48.9(3)	1.03

For comparison, in Figure 9 we plot the ^57^Fe ZF-NMR spectrum of maghemite nanoparticles of about 10 nm in diameter measured at 4.2 K in [27] (blue dashed line) along with our spectra. The qualitative similarity of the two experimental spectra is obvious, especially with regard to the position and width of the main narrow peaks.

The low frequency narrow peak at 70.92 MHz in the spectra of our nanoparticles (Figure 9) seems to correspond to iron ions in the tetrahedral A-site and the high frequency peak corresponds to iron ions in the octahedral B-site nuclei. This result correlates with the Mössbauer data and is in agreement with the data observed in [17] for maghemite.

The only feature of the experimental ^57^Fe ZF-NMR spectra of our nanoparticles that remains unexplained is the low-frequency broad shoulder (Figure 9). On the one hand, it could be related to a specific size distribution of nanoparticles. On the other hand, this part of spectrum could originate from Fe nuclei situated on the surface of the nanoparticles. In the latter case, it is not difficult to explain the absence of a low-frequency shoulder in the ^57^Fe ZF-NMR spectrum of maghemite particles, as reported in [27–28]. Indeed, the diameter of our nanoparticles is about 5–8 nm, which is almost two times less than that of the nanoparticles studied in [27–28], and hence, the partial amount of the surface Fe atoms *N*_surface_/*N*_volume_ ~ *d*^2^/*d*^3^ ~ 1/*d* is also two times higher. Moreover, in [12], the core–shell structure iron oxide nanoparticles of the same size has already been described and calculated based on magnetic measurements.

In the HSA-coated sample, we also succeeded to observe the ZF-NMR signal, despite the very low iron content per unit volume and the resulting low signal-to-noise ratio. As evident from Figure 9, at least at 4.2 K, there is no significant difference in the NMR spectra of the HSA-coated and uncoated samples.

## Conclusion

In this work, we studied and compared the structural and magnetic properties of uncoated and functionalized (HSA-coated) iron oxide nanoparticles by employing XRD, TEM, Raman, ^57^Fe ZF-NMR and Mössbauer spectroscopy. The TEM and XRD measurements revealed the spherical shape of the nanoparticles with an average diameter of 5–8 nm and a cubic spinel-type crystal structure (space group 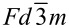) corresponding to the Fe_3_O_4_ or γ-Fe_2_O_3_ phase. The Raman, Mössbauer and NMR spectra clearly indicated the presence of the γ-Fe_2_O_3_ phase. Moreover, Mössbauer spectroscopy revealed the different magnetic behavior of uncoated and HSA-coated nanoparticles. This can result from the weakening of the magnetic interactions between the nanoparticles in the coated sample due to the separation of the MNPs by nonmagnetic HSA protein molecules. In particular, this leads to the transition of coated nanoparticles to the paramagnetic state at a lower temperature. The NMR spectra of uncoated, functionalized nanoparticles revealed no changes in the hyperfine parameters of the ^57^Fe nuclei. We show that ^57^Fe ZF-NMR spectroscopy on iron oxide nanoparticles with natural ^57^Fe abundance allows for the clear identification of the high frequency peaks corresponding to the A- and B-sites in the crystal structure of γ-Fe_2_O_3_. In addition, we observe a broad shoulder in the lower frequency region of the NMR spectrum, which most likely originates from iron atoms located in the surface layer of the particles. This assumption is indirectly confirmed by the absence of such a shoulder in the spectra of similar particles of larger size, which thus have a smaller ratio of surface/core atoms [27–28]. The obtained results are important for understanding the structural and magnetic properties of iron oxide nanoparticles used as *T*_2_ contrast agents for MRI.

## Experimental

### Synthesis of the nanoparticles

Uncoated iron oxide nanoparticles were synthesized by the thermal decomposition of iron(III) acetylacetonate. Briefly, the solution of iron(III) acetylacetonate (2 g) in anhydrous benzyl alcohol (40 mL) was heated to 110 °С for 1 h. Afterwards, the solution was heated to reflux under nitrogen atmosphere and kept for 30 min under reflux. Subsequently, the solution was cooled down, and the MNPs were precipitated by acetone, then thoroughly washed with acetone and dried. HSA-coated MNPs were synthesized by the following procedure. First, the MNPs (80 mg) were placed in NaOH aqueous solution (30 × 10^−3^ M, 20 mL) and the mixture was stirred until all the precipitate was suspended. After that, an HSA solution (40 mg mL^−1^, 20 mL) was added. The resulting mixture was stirred for 15 min and filtered through syringe filters with a pore diameter of 0.2 μm (Millipore, USA) to remove any large particle agglomerates. After overnight dialysis against distilled water (25 kDa), NaOH (1 M, 1 mL) was added to the nanoparticle suspension. Then, 920 μL of glutaraldehyde was added and the mixture was incubated for 15 min with constant stirring. After that, the reaction was stopped by adding a glycine solution in water (3 M, 1 mL, pH 9.2), followed by stirring for 1 h. Finally, a NaBH_4_ solution in phosphate-buffered saline (10 mg mL^−1^, 1.3 mL) was added to the reaction mixture that was incubated for another 2 h. To remove excess low molecular weight substances and unbound molecules of HSA, the final solution was washed with distilled water on 100 kDa MWCO centrifugal filters (Amicon, USA) until the filtrate became colorless. The solution of HSA-coated MNPs was sterilized through 0.22 μm sterile filters, lyophylized and stored under room temperature [29]. The applicability of these nanoparticles as a contrast agent in MRI was previously demonstrated on the experimental rat C6 glioma model [7].

### Experimental methods

TEM of pure and HSA-coated MNPs was performed using a Titan 80-300 TEM/STEM (ThermoFisher Scientific, USA) operated at 300 kV. XRD spectroscopy was carried out with a Rigaku MiniFlex diffractometer using Cu Kα radiation. The Mössbauer absorption spectra of ^57^Fe nuclei were recorded in the temperature range of 10–300 K with a standard MS-1104Em spectrometer operated in the constant acceleration regime [30–31]. The gamma ray source ^57^Co(Rh) was maintained at room temperature. The calibration was performed with a metal α-Fe standard absorber. The XRD patterns were collected using the Rigaku MiniFlex equipment of the Shared Research Center FSRC “Crystallography and Photonics” RAS. Cu Kα radiation (λ = 0.154 nm) was used.

The Raman spectra were recorded at room temperature with a 671 nm laser as the excitation source. A Princeton Instruments Acton SP2500 monochromator/spectrograph equipped with a Spec-10 system and a nitrogen-cooled CCD detector was used to collect the spectra. The laser power at the sample was ≈0.5 mW.

The ^57^Fe ZF-NMR spectra were measured at 4.2 K with a home-built, phase coherent, pulsed NMR spectrometer using a frequency step, point-by-point, spin echo technique. The ZF-NMR spectra were obtained by integration over the spin echo magnitude envelope in the time domain at a given frequency and averaging over the scan accumulation number. Due to the low natural abundance of the ^57^Fe isotope (2.19%), a relatively large amount (about 1.5 g) of powder Fe nanoparticles was used in our ZF-NMR experiment.
